# Adolescents’ academic performance: what helps them and what hinders them from achievement and success?

**DOI:** 10.3389/fpsyg.2024.1350105

**Published:** 2024-07-10

**Authors:** Simona Horanicova, Daniela Husarova, Andrea Madarasova Geckova, Miriama Lackova Rebicova, Lenka Sokolova, Andrea F. de Winter, Sijmen A. Reijneveld

**Affiliations:** ^1^Department of Health Psychology and Research Methodology, Faculty of Medicine, PJ Safarik University, Košice, Slovakia; ^2^Olomouc University Social Health Institute, Palacky University in Olomouc, Olomouc, Czechia; ^3^Department of Community and Occupational Health, University Medical Center Groningen, University of Groningen, Groningen, Netherlands; ^4^Institute of Applied Psychology, Faculty of Social and Economic Sciences, Comenius University in Bratislava, Bratislava, Slovakia

**Keywords:** adolescents, academic performance, academic success, adolescents’ experiences, qualitative study

## Abstract

**Introduction:**

Research on adolescents’ academic performance has mostly focused on the contribution of objective factors, such as socioeconomic situation of the family or individual cognitive skills and school results. Evidence with a focus on adolescents’ subjective experiences is scarce. The aim of this qualitative study was to explore factors related to adolescents’ academic performance from their perspectives.

**Methods:**

We used data from 11 group semi-structured interviews conducted in 2020/2021 with 45 adolescents in the first year of high school in Slovakia (mean age = 14.98; 22.2% boys). Participants were selected from three types of high school with regards to the graduation system. We analysed the data using consensual qualitative research and thematic analysis.

**Results:**

Based on the statements of the adolescents, we identified five main themes of factors that affect their academic performance. Adolescents reported that the following contribute to their academic performance: the contents and methods of teaching; how teachers behave and do their jobs; the way in which adolescents study and what motivates them; support within and outside the school, and the environment and appearance of the school. Adolescents reported that improving the curricula and using teaching methods that balance theoretical information with practical skills training would help their academic performance immensely.

**Conclusion:**

We identified several factors related to adolescents’ academic performance using their own perspectives and experiences. Strengthening the capacities of teachers may largely benefit adolescents’ educational process and further academic performance.

## 1 Introduction

Academic performance is an important indicator of adolescents’ academic success and achievement (Kumar et al., 2021). Previous research indicates that adolescents are more likely to perform better on standardised achievement tests and have higher grade averages and standardised test scores, when they are engaged and participate actively in school. That regards school behaviour (i.e., attending school regularly, following school rules, concentrating on learning), feeling connected and belonging to school, having positive feelings toward teachers and peers, and using strategic approaches to learn (Wang and Holcombe, 2010). Showing such engagement and active participation in academic activities and tasks is associated with the intrinsic motivation for school and with the school satisfaction of adolescents (Libbey, 2004; Magnano et al., 2020).

According to the international Health Behaviour in School-aged Children (HBSC) study, only one third of young adolescents in Slovakia is satisfied with school and this number becomes even lower with increasing age (Madarasová Gecková and Husarova, 2023). Slovakia has also been ranked very low regarding the degree to which adolescents like school for over a decade compared to the other European countries (Inchley et al., 2016, 2020). However, a considerable number of the adolescents who report not liking school still care about the education they will achieve (Bosakova et al., 2020; Horanicova et al., 2020). These findings imply that the demand for education is evident, but that the actual education does not meet the needs and perspectives of adolescents.

Academic performance of adolescents is affected by the social and cultural capital inherited from the family (Denzler, 2011; Fan, 2014). Parental education levels and socioeconomic situation may affect educational resources, opportunities and the academic trajectory of their children (Fan, 2014). The study by Williams et al. (2019) emphasised crucial role of school support for adolescents from low-income background, including establishing meaningful parent-school collaboration or creating culture of hope for a better future, to help them deal with challenges and support their motivation for academic achievement and success. Moreover, congruence between adolescents and their parents’, especially mothers’, wishes regarding career choice, has an important impact on academic motivation, work hope and occupational importance, which in turn has a positive effect on future intentions to undertake university studies (Fantinelli et al., 2023). This was also supported by another study, where adolescents expressed that their personal educational goals and beliefs closely align with those communicated by their mothers (Suizzo et al., 2016). Academic performance reflects adolescents’ potential to learn and prosper (Farooq et al., 2011). Adolescents who perform well at school and experience academic success are more likely to continue with higher education, apply for and secure better jobs and higher salary for themselves and to become fully fledged contributors to society (Ashby and Schoon, 2010; Olshansky et al., 2012). Better individuals’ socioeconomic position and social capital positively influence the ability to access healthcare and decide on health-promoting options, and thus enhances overall health. In this way, may alleviate the current strain on the social system (Abel, 2008). On top of that, academic performance may impact the socioeconomic development of a country overall (Singh et al., 2016).

The main determinants of students’ academic performance have previously been shown to result from the educational process (Narad and Abdullah, 2016; Singh et al., 2016). On top, there is a range of psychological factors which might positively or negatively influence academic performance, including adolescents’ motivation, level of stress and capacity to cope with it, anxiety, self-esteem or self-efficacy. It seems that intrinsic motivation and self-efficacy are the key aspects (Tindle et al., 2022). A scoping review of Honicke and Broadbent (2016) showed that students with higher level of self-efficacy are more motivated to learn and apply more effective learning strategies what lead to better performance at school. Katsantonis and McLellan’s (2023) qualitative study also highlighted the significance of motivation and self-regulated learning strategies in academic achievement. It showed that adolescents use various task-specific and general self-regulated strategies, such as organization and time management, along with their motivational beliefs and academic emotions (e.g., interest, enjoyment, satisfaction), to achieve good grades and enhance schoolwork engagement (Katsantonis and McLellan, 2023). Other relevant factors regard individual characteristics of the students, such as cognitive and intellectual skills, learning styles, skills abilities, or personality (Lu et al., 2011; Miñano et al., 2012; Kumar et al., 2021). Research has shown that academic hardiness as important personality characteristic help adolescents effectively manage their school failures, cope with negative academic experience, such as low grades in the test, and transform it into opportunity for growth (Kamtsios and Karagiannopoulou, 2013). Social environmental factors have an important influence on academic results, as well. Besides family socioeconomic position and parental education level, having good relationships of adolescents with peers, parents, and teachers improves the academic outcomes, performance, and achievement of these adolescents (Goddard, 2003; Farooq et al., 2011; OECD, 2018). Adolescents as students are themselves the main protagonists of the educational process and their own academic trajectory. Gaining information from their perspective on what may contribute to their performance and success at school may be significantly add to identifying the main determinants of academic success and performance.

To sum up, academic performance in connection with a wider range of determinants or consequences has been studied, but most research is quantitative and research on subjective experiences and perspectives from adolescents’ point of view lacks. A qualitative approach allows us to investigate academic performance more deeply and to obtain a closer look at all possible factors that affect it. Therefore, the aim of this study was to identify the main factors that help and hinder adolescents in their performance at school from the perspective of the adolescents themselves.

## 2 Methods

### 2.1 Design of the study

We conducted a qualitative study embedded in the international HBSC (Health Behaviour in School-aged Children) study on the health-related behaviour, well-being and social context of adolescents.^1^ The study was conducted using semi-structured group interviews and the data were analysed by consensual qualitative research (CQR) methods and inductive thematic analysis (Braun and Clarke, 2022). The CQR methodology involves in the research process the diversity of experiences and opinions of each member of the research team and their ability to reach conclusions with regards to their assumptions (Hill et al., 2005). A diversity of experiences was obtained by selecting members of the research team from different backgrounds, including health psychology, school psychology and social work. Data-driven thematic analysis is used to identify and analyse topics and themes across the analysed qualitative data using the different experiences and backgrounds of each member of the research team (Braun and Clarke, 2006).

### 2.2 Study setting, sampling and participants

The target population for the study comprised adolescents in the first year of Slovak high school, age 15–16 years. We conducted 11 group interviews with 45 participants in total (10 boys; mean age = 14.95). The participants were selected from three types of high schools in Košice, Slovakia; i.e., grammar schools (with A levels graduation), secondary schools with GCSE graduation and secondary schools with apprenticeship certificate graduation. We contacted participants in several steps. First, we got in touch with the school administrators to inform them about the study and ask them to participate. After obtaining their consent, we contacted the parents of the participants with the help of teachers and obtained parental informed consent. Thereafter, we approached the participants and obtained their informed consent. All participants were informed about the voluntary participation in the study and anonymity of the provided data and were allowed to withdraw from the study at any time.

The study was conducted in accordance with the guidelines of the Declaration of Helsinki (1964) as well as with the consolidated criteria for reporting qualitative research (COREQ) (Tong et al., 2007). It was reviewed and approved by the Ethics Committee of the University (19N/2020).

### 2.3 Procedure and measures

The data collection occurred in several steps. First, adolescents were asked to fill in a questionnaire created by the authors that regarded the questions of age, gender, size of the place of residence, their liking of school and attitude toward education. These questionnaire data were used to describe the sociodemographic characteristics and the level of school satisfaction (combination of liking school and attitudes toward education). The measures regarded standardised questions used in HBSC data collection. Liking school was measured using the item: “How do you feel about school at present?,” with four-point Likert-type responses (“I like it a lot”; “I like it a bit”; “I do not like it very much”; “I do not like it at all”). Attitudes toward education were measured using the item “Do you care what kind of education you will have?,” with three-point Likert-type responses (“I care a lot”; “I care about it, but not too much”; “I could not care less”) (Madarasová Gecková, 2019). Next, we asked the adolescents to participate in an online interview and then conducted semi-structured group interviews between November 2020 and June 2021. The interviews were recorded on video. Finally, we transcribed the video records, leaving out any personal information of the participants. The length of the interviews was between 45 and 60 min.

We conducted the interviews during the second wave of the COVID-19 pandemic in Slovakia, a time in which face-to-face contact with participants was not feasible due to anti-pandemic measures. We therefore conducted the interviews using the Zoom online platform. The interviews were led by a trained professional in psychology who has experience working with young adolescents in an online counselling platform. The members of the research team (AMG, DH, MLR, LS, SH) were all professionals with different background and experience in psychology and social work, and all of them participated in the interviews as silent observers.

The interview questions were selected to extend the quantitative findings of the international HBSC study. These results showed that a considerable number of adolescents in Slovakia reported a low level of school satisfaction, but that they also care about their future education (Horanicova et al., 2020; Madarasová Gecková and Husarova, 2023). Important factors contributing to their positive attitudes toward school were relationships with teachers and schoolmates and schoolwork support inside or outside the family, especially in adolescents from low and middle SES (Horanicova et al., 2020, 2022). Based on these findings and in line with the aim of the study we formulated the following questions:

1. Some children like school and other children do not – why do you think that is? Why do some children like being at school and others do not?
2. Many children feel that their teachers care about them. How do you know that your teacher truly cares about you?
3. Some children are quite successful at school and others not as much. How do we help those who struggle at school?
4. What should school, its teachers, students or even people from outside the school do in order to create a safe and pleasant environment?
5. What should be done (by teachers, students, outsiders) so that the time children spend in school is effectively used?

### 2.4 Data handling and analyses

In the data handling, we processed the data obtained from the segments of the transcripts that referred to academic performance and its contributors, to extract codes that we could further analyse. We did so by first transcribing the interviews verbatim in the Slovak language, checking the transcripts to ensure their accuracy and uploading them into the MAXQDA standard platform for data analyses (VERBI Software, 2021). Inductive, data-driven thematic analysis (Braun and Clarke, 2022) was used to analyse the data. All members of the research team were researchers trained in CQR. The coders first familiarised with the data individually. After carefully reading each transcript, each member of the team coded the segments of the transcript and generated the initial codes. Next, all of the members met for cross-checking and evaluation of the generated codes and coded segments of each transcript. In case of different opinions, discussion continued until a consensus was reached. The final code book was again revised by the coders and newly generated or reformulated codes were applied to the previously coded segments.

In the analyses, we assessed the main factors that help and hinder adolescents in performing at school, using a thematic analysis of the codes produced in the data handling. We did so by clustering the codes into themes and subthemes, once again done by all members of the team individually. Afterwards, the team members met for cross-checking of the created themes and subthemes, and a discussion was held until a consensus on the final thematic map was reached.

## 3 Results

### 3.1 Characteristics of the sample

Table 1 provides descriptive characteristics of the sample. Almost half of the participants attended grammar school; the other half of the participants attended secondary schools graduating with GCSE or apprenticeship certificate. Most of the participants reported feeling satisfied toward school and education (like school, care about education). Almost a third of the participants reported feeling inconsistent with school and education (do not like school, care about education).

**Table 1:** Descriptive characteristics of the sample (*N* = 45).

Characteristic	Number
Gender	
Boys	10
Girls	35
Type of school	
Grammar school	22 (13 girls, 9 boys)
Secondary school (GCSE)	20 (girls)
Secondary school (apprenticeship certificate)	3 (2 girls, 1 boy)
School satisfaction*	
Satisfied	32
Inconsistent	13
Age distribution	
13 years old	1
14 years old	10
15 years old	23
16 years old	11

*Satisfied – like school, care about education. Inconsistent – do not like school, care about education.

### 3.2 Promoting and hindering factors

**Figure 1:**
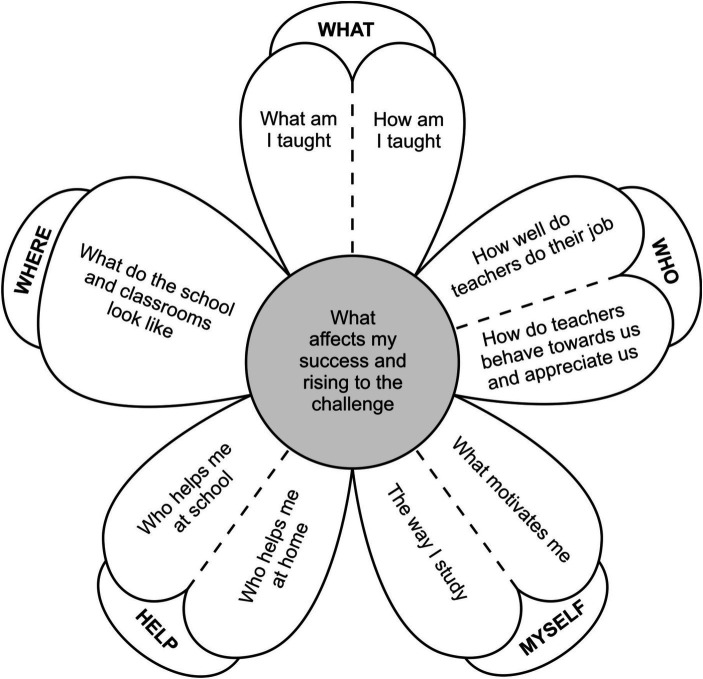
Main factors contributing to adolescents’ academic performance.

The thematic analysis led to a model consisting of the factors that hinder or help adolescents in their academic performance and in overcoming challenges in their learning process. The model is depicted in Figure 1. The statements of adolescents imply that they do not see themselves as contributors to their own achievement and success to a great extent. On the contrary, they reported external factors to be far more influential in the trajectory to adolescents’ achievements. The factor reported to have the greatest impact was the role of teachers, including relationships with students and the way they teach. The teaching process depends on what is being taught and the way it is taught. The physical environment and equipping of the school and classes limit/facilitate the teaching process and methods of teaching into some extent. Students’ relationships with teachers are important and affect their motivation. Adolescents reported using other available sources of support with learning and studying as well, but only after the primary role of the teacher as a provider of education fails.

#### 3.2.1 Main themes

We identified five main themes regarding factors that contribute to adolescents’ academic performance: *What, Who, Myself, Help, Where.* The themes are depicted in Figure 1 and will be further described in more detail.

##### 3.2.1.1 What

Adolescents reported that the contents and methods of teaching significantly affect their achievement and learning process. One of their main concerns was the lack of emphasis on gaining practical skills and by contrast too much emphasis on memorising excessive amounts of information, which adolescents consider unnecessary without any added value or applicability. Moreover, replacing a passive teaching style with active methods of teaching that would enable adolescents to gain new, interesting and useful information while learning as much as possible during school hours would help them immensely.

“… So, using a few more practicalities. Perhaps, during Biology, we could conduct more experiments or search for evidence or something like that. Because many times during Maths, the teacher would just solve a problem and would say ‘I’m not in a mood to prove it; you just have to believe me that this is the way it is supposed to be.’ So, providing more evidence, because it’s more interesting to me then ….”

##### 3.2.1.2 Who

The factor most often mentioned by adolescents regarding their academic performance was the teacher. Moreover, adolescents declared that teachers alone are mostly responsible for their behaviour and building their relationships with students. A teacher who makes an effort to prepare for class and teach effectively and a teacher who is willing to give a student a second chance, adjust to the individual needs of students and develop their potential is most likely capable of inspiring and leading students to success. Additionally, a teacher who is able to lighten the atmosphere while maintaining authority and treat students with respect and fairness is capable of cultivating healthy relationships with students. Finally, a teacher who is not interested in doing their job, treats students unjustly and is not willing to accept individual differences of students’ comprehension may discourage students from further achievement.

“Well, I think that a second chance from a teacher is a kind of thing that makes me happy and when I see that they aren’t indifferent about me getting a bad grade and they give me a chance to fix it… The second chance is really important….”

##### 3.2.1.3 Myself

Adolescents mentioned themselves as a determinant of their performance the least frequently. Adolescents reported that their own ability to stay focused, find their own way of effective studying, manage their time and seek support with studying may help them perform better to some extent. Moreover, adolescents reported being motivated by all sorts of incentives, such as setting long-term goals and experiencing success on the one hand and, interestingly, on the other hand by failure, bad grades, fear of teachers and avoiding being punished by their parents.

“Well, I think that if students are successful, they attend school with more joy and they are happy to explore and gain new experiences, and if they experience failure, they lose motivation… ‘Why should I learn this and that…’ and they often are more truant.”

##### 3.2.1.4 ‘Help’

Adolescents indicated that support available within and outside school contributed to their performance. Within school mutual help and schoolwork support, such as extra dedicated teaching time from teachers and peer support are additional sources of necessary support that help adolescents prosper. Outside school this included conditions for studying at home and support from parents and other experts in their field of expertise who are willing to help.

“I would say that we were a very small class during elementary school. Five of us began ninth grade and our relationships were like, if someone was less successful, we would always get together and try to explain everything as best as we could. We would help each other all the time. The relationships in a classroom are very important… When we are able to help and support each other.”

##### 3.2.1.5 Where

Adolescents also reported the physical environment at school to be an important factor related to their performance. They reported that old or worn-out classrooms, insufficient and outdated school equipment, a lot of noise and a lack of study space inhibit an effective teaching process and further achievement of adolescents.

“I think that the teaching environment is very important. They feel much better in new classrooms, not in those where the plaster is falling down. And the teachers do a lot that affect them as well. And for example, using old books for studying… They should be taught in a new way, not the way from 20 to 30 years ago. Thus, this affects the environment and comfort in classroom and teaching process as well.”

Table 2 provides more examples of narratives of the adolescents within all five themes.

**Table 2:** Quotes illustrating factors related to adolescents’ academic performance.

Themes/*Subthemes*	Quotes
WHAT	
*What am I taught*	*“… I would say that we are taught a lot of theory, but we are not able to say a common definition. … So, using a few more practicalities. Perhaps, during Biology, we could conduct more experiments or search for evidence or something like that. Because many times during Maths, the teacher would just solve a problem and would say ‘I’m not in a mood to prove it; you just have to believe me that this is the way it is supposed to be.’ So, providing more evidence, because it’s more interesting to me then …”*
*How am I taught*	*“When the teacher is capable of making a lesson more interesting, when it’s not just about the same-old writing notes on the board but when they try to engage students and make it more interesting for them.”* *“Well, the teacher who taught me this did it in a playful way. She was able to divide the topic and if we did not understand, we could just ask her and she explained it using real life examples. And the other teacher who taught me in a way I did not understand just rushed through the topic really quickly, even though we told her we did not understand … She did not have a way of teaching that we needed.”*
WHO	
*How well do teachers do their jobs*	*“Well, I think that a second chance from a teacher is a kind of thing that makes me happy and when I see that they aren’t indifferent about me getting a bad grade and they give me a chance to fix it… The second chance is really important…”* *“Or when they repeat something twice, because they make an effort in order to help us understand.”* *“Yes, a second chance, because we are all humans. Not everything works out for everyone all the time and teachers make mistakes, too, so I think a teacher providing a second chance is very important, but not always, you know. Because we would take it for granted.”*
*How teachers behave toward us and appreciate us*	*“Simply, teachers should praise some students even for the smallest things, because it may be enough for some to think that they did something for themselves and made progress.”* *“So that they do not discriminate between the students. Because I know they may find their favourite one, and they would do everything for them, but then other students are the worst and are treated different way than their favourite ones.”*
MYSELF	
*What motivates me*	*“Well, I think that if students are successful, they attend school with more joy and they are happy to explore and gain new experiences, and if they experience failure, they lose motivation… ‘Why should I learn this and that…’ and they often are more truant.”*
*The way I study*	*“When I have a problem understanding something, I just ask the teacher, whether she would be willing to explain it to me one more time and if I did not ask a teacher, I would, I do not know, call my classmate or my sister or if I knew someone older at school who understands this, I would ask them for help”* *“Well, focus and pay attention for sure. Do not look out the window to see how many cars have passed but really focus and pay attention to the teacher and do everything that is necessary, the exercises, homework, because it can be really useful; you do not need to go over it again because you understand it, because you were paying attention.”*
HELP	
*Who helps me at home*	*“Perhaps parents play a role in this, too. I remember my friend; her father is a lawyer and he’s really good at history. So if her dad teaches her history in a different way than her teacher, she can work with that. Parents play a big role in my opinion.”*
*Who helps me at school*	*“I would say that we were a very small class during elementary school. Five of us began ninth grade and our relationships were like, if someone was less successful, we would always get together and try to explain everything as best as we could. We would help each other all the time. The relationships in a classroom are very important… When we are able to help and support each other.”*
WHERE	
*What do the school and classrooms look like*	*“I think that the teaching environment is very important. They feel much better in new classrooms, not in those where the plaster is falling down. And the teachers do a lot that affect them as well. And for example, using old books for studying… They should be taught in a new way, not the way from 20 to 30 years ago. Thus, this affects the environment and comfort in classroom and teaching process as well.”*

## 4 Discussion

The aim of this study was to identify the main factors hindering and promoting with the academic performance of adolescents using the perceptions and experiences of the adolescents themselves. Five main themes of factors that help and hinder adolescents’ academic performance were identified based on the statements of the adolescents. Adolescents reported that influences contributing to their academic performance include the contents and methods of teaching as well as the way the teachers behave toward them and how they do their jobs. Our results suggest that the way adolescents study, their sources of motivation, support within and outside school and the overall environment and appearance of the school affect their academic performance, as well.

We found that adolescents considered the external factors at school more influential than their own impact on their academic performance. The most frequently mentioned factor which determines adolescents’ academic performance was the role of teachers. Bolstering teachers’ skills and their relationships with students may considerably improve adolescents’ academic performance. These findings are in line with previous research (Asikhia, 2010). Teachers who are competent in doing their job, prepared for class, inspiring and thorough during the teaching process are perceived as facilitators in students’ performance at school, and this is supported by previous research (Adu and Olatundun, 2007). Moreover, teachers who are willing to give students a second chance, respond to their individual needs and develop students’ potential thus help adolescents prosper on their academic journey, which also corresponds with previous research focused on students’ satisfaction with positive behaviours of their teachers (Shah, 2009). This becomes essential especially in situations when assessment practices create pressure on adolescents and make them anxious what might subsequently impact their achievement in negative way (Katsantonis and McLellan, 2023).

Additionally, we found that teachers who are able to maintain their authority while keeping the atmosphere casual and who treat students fairly and with respect are likely to improve adolescents’ motivation and further academic performance. These findings support previous research findings that students’ academic performance depends on teachers’ competence skills and knowledge of the subject matter (Adediwura and Tayo, 2007). Furthermore, previous studies have shown that teachers with positive attitudes who show patience and care about their students’ needs help improve students’ performance at school (Adediwura and Tayo, 2007). According to students’ experiences, a warm and caring student-teacher relationship – one that creates a safe atmosphere, fosters well-being, and provides academic help with schoolwork – has a significant influence on improving their achievement as well as behaviour (Jeffrey et al., 2013). Teachers are one of the main contributors to the teaching process and an important factor influencing adolescents’ academic performance and motivation. Teachers’ competence, attitudes and behaviour may affect adolescents’ achievement and subsequently their further academic journey. Thus, the perspectives of adolescents align with findings from previous research.

We found that too much memorising of inapplicable information, a lack of practical skills and using passive methods of teaching seem to significantly hinder adolescents’ achievement and performance. This is in line with previous findings that, in contrast, using active teaching methods while focusing on new, interesting and valuable information allows adolescents to develop practical and useful skills and improve their academic performance (Brame, 2016). Teaching in a way that allows gaining new information along with promoting critical thinking and interest rather than demanding simple rote learning has proven to be an effective way of educating (Hesson and Shad, 2007; Ganyaupfu, 2013). Moreover, from adolescents’ perspective, providing feedback on their progress and learning along with an engaging and stimulating teaching style is considered important effective learning and ultimately leads to academic success (Katsantonis and McLellan, 2023). The contents of curricula and teaching methods are important factors contributing to adolescents’ academic performance. Taking them into an account with regard to the design of syllabi may help adolescents’ further academic journey and success.

We found that the physical environment of school and classrooms along with insufficient and old school equipment impairs effective teaching processes and the performance of adolescents. These findings support previous ones showing the importance of an adequate physical environment at school and in classrooms, which may affect adolescents’ motivation, results, performance and achievement (Suleman and Hussain, 2014; Usaini et al., 2015; Edgerton and McKechnie, 2023). Moreover, the study of Person et al. (2016) has shown that changes in the physical school environment can from adolescents’ point of view significantly influence their school satisfaction and peers’ relationships by either facilitating or hindering opportunities for socialization, communication, and connection among them. The physical environment and appropriate equipment at school are thus important factors affecting teaching and the learning processes of adolescents and may facilitate their further performance.

We found that mutual help along with support with studying at school and conditions at home affect adolescents’ academic performance. This is in line with previous research showing that a helpful atmosphere in classrooms and cooperation can importantly improve adolescents’ engagement and efficacy (Dorman, 2001; Wang and Holcombe, 2010), which may subsequently increase their success and performance at school. An overall positive psychosocial climate in a class, which includes clear norms and values among schoolmates, significantly contributes to strengthening adolescents’ relationships and mutual support. This, in turn, enhances adolescents’ school satisfaction and improves their performance (Person et al., 2016, OECD, 2018). Moreover, support with homework at home improves adolescents’ motivation and engagement (Daw, 2012; Moé et al., 2018), which may influence their further achievement. Involving significant others, such as parents or siblings in learning process can have a positive impact on adolescents’ learning outcomes. By fostering a supportive environment and encouraging active help-seeking behaviour, adolescents can develop the necessary skills and motivation to be academically successful and achieve their goals (Katsantonis and McLellan, 2023). Available support within school and outside of school seems to be an important addition to adolescents’ academic performance, particularly when the primary role of teachers as educators is inefficient.

Our results show that adolescents do not perceive themselves as a strongly influential factor in their own academic performance. These findings align with previous research showing that adolescents do not consider themselves as a relevant contributor to their school experience; it is thus important to facilitate their engagement and performance from an early age (Boberova et al., 2017). We found that adolescents’ ability to focus and pay attention and their ability to find a preferred way of studying and managing their own time are helpful factors influencing their academic performance. Moreover, adolescents reported several incentives that keep them motivated to perform better at school, including long-term goals and experiencing success but also failure, fear of teachers and avoiding punishment. These issues appear to be understudied, although some studies have shown that academically motivated students are willing to show higher efforts for studying and self-regulation in learning strategies, which subsequently affects their academic performance (Pintrich, 2003; Ariani, 2016). From our interviews, we learned that with regard to their own academic performance, adolescents perceive the influence of their own contributions to be relatively small.

### 4.1 Strengths and limitations

One of the main strengths of this qualitative study is the detailed exploration of the factors affecting adolescents’ academic performance using their own perceptions and experiences. Moreover, using consensual qualitative research helped us avoid the individual subjective perspectives of the researchers, since every member of the research team had to agree on the codes used in coding of the data. A limitation of this study is that we may not have reached a heterogeneity in the sample representative of the apprenticeship graduation students. However, during data collection, we reached a point of saturation when no new themes appeared. Another limitation is that the interviews were conducted during the second wave of the COVID-19 pandemic in Slovakia. Although we aimed to focus adolescents on their experiences before the pandemic during the interviews, it is likely that their perspectives were still influenced in some extent by the restrictions related to school attendance.

### 4.2 Implications

The findings of this study have several implications for practice. First, designing curricula should be focused on balancing useful theoretical information with obtaining practical and applicable skills and using appropriate teaching methods in order to facilitate the transfer of the information to students in the most effective way.

Second, teachers’ behaviour, their approach to teaching and their knowledge and relationships with students play an important role in adolescents’ academic journey. Strengthening teachers’ competence and skills and providing them with appropriate complementary training may facilitate the teaching process and improve their relationships with students, which may significantly help adolescents’ achievement and further success.

Next, providing adolescents with additional support with schoolwork within or outside of school in the form of tutoring from experts in certain fields may help them overcome the struggles they experience with studying. Additionally, the renovation of classrooms and equipment and investment in new books and teaching aids should be encouraged in order to create a pleasant and stimulating teaching environment.

The findings of this study also provide some implications for future research. More evidence is needed on the learning style mechanisms of adolescents and management of their responsibilities, as well as the role of teachers in facilitating this process. In addition, intervention research in the school environment focused on self-efficacy and school performance of adolescents might deepen understanding of this relationship.

## 5 Conclusion

We identified five themes regarding factors contributing to adolescents’ academic performance: 1. What, 2. Who, 3. Myself, 4. ‘Help,’ 5. Where. Bolstering teachers’ expertise and approach toward students in order to improve the teaching process and overall atmosphere has been raised. Additionally, enhancing the school’s physical environment and equipment is an important factor contributing to adolescents’ academic performance.

## Data availability statement

The raw data supporting the conclusions of this article will be made available by the authors, without undue reservation.

## Ethics statement

The studies involving humans were approved by the Ethics Committee of the PJ Safarik University. The studies were conducted in accordance with the local legislation and institutional requirements. Written informed consent for participation in this study was provided by the participants’ legal guardians/next of kin.

## Author contributions

SH: Conceptualization, Formal analysis, Investigation, Methodology, Project administration, Writing – original draft. DH: Conceptualization, Formal analysis, Methodology, Project administration, Supervision, Validation, Writing – review & editing. AM: Conceptualization, Formal analysis, Methodology, Supervision, Writing – review & editing. ML: Conceptualization, Formal analysis, Investigation, Writing – review & editing. LS: Conceptualization, Formal analysis, Methodology, Supervision, Writing – review & editing. AW: Conceptualization, Methodology, Supervision, Validation, Writing – review & editing. SR: Conceptualization, Methodology, Supervision, Validation, Writing – review & editing.

## Footnotes

^1^more information on www.hbsc.org.
